# Characteristics of the pulmonary opacities on chest CT associated with difficulty in short-term liberation from veno-venous ECMO in patients with severe ARDS

**DOI:** 10.1186/s12931-023-02425-2

**Published:** 2023-05-10

**Authors:** Mitsuaki Nishikimi, Shinichiro Ohshimo, Wataru Fukumoto, Tatsuhiko Anzai, Kazuo Awai, Takayuki Ogura, Toshikazu Abe, Mamoru Masuda, Kenji Fujizuka, Mitsunobu Nakamura, Michihito Kyo, Kunihiko Takahashi, Nobuaki Shime

**Affiliations:** 1 Department of Emergency and Critical Care Medicine, Graduate School of Biomedical and Health Sciences, Hiroshima University, 1-2-3 Kasumi, Minami-ku, Hiroshima, 734-8551 Japan; 2 Department of Emergency and Critical Care Medicine, Nagoya University Graduate School of Medicine, Nagoya, Japan; 3 Department of Diagnostic Radiology, Graduate School of Biomedical and Health Sciences, Hiroshima University, Hiroshima, Japan; 4 Department of Biostatistics, M&D Data Science Center, Tokyo Medical and Dental University, Tokyo, Japan; 5 Department of Emergency Medicine and Critical Care Medicine, SAISEIKAI Utsunomiya Hospital, Utsunomiya, Japan; 6 Department of Emergency and Critical Care Medicine, Tsukuba Memorial Hospital, Tsukuba, Japan; 7 Department of Health Services Research, Faculty of Medicine, University of Tsukuba, Tsukuba, Japan; 8 Advanced Medical Emergency Department and Critical Care Center, Japan Red Cross Maebashi Hospital, Maebashi, Japan

**Keywords:** ARDS, ECMO, Pulmonary opacity, Mixed pattern of pulmonary opacities, Signs of traction bronchiectasis

## Abstract

**Background:**

It is clinically important to predict difficulty in short-term liberation from veno-venous extracorporeal membrane oxygenation (V-V ECMO) in patients with severe acute respiratory distress syndrome (ARDS) at the time of initiation of the support. The aim of this study was to identify the characteristics of pulmonary opacities on chest CT that is associated with difficulty in short-term liberation from V-V ECMO (< 14 days).

**Methods:**

This multicenter retrospective study was conducted in adult patients initiated on V-V ECMO for severe ARDS between January 2014 and June 2022. The pulmonary opacities on CT at the time of initiation of the ECMO support were evaluated in a blinded manner, focusing on the following three characteristics of the opacities: (1) their distribution (focal/diffuse on the dorso-ventral axis or unilateral/bilateral on the left-right axis); (2) their intensity (pure ground glass/pure consolidation/mixed pattern); and (3) the degree of fibroproliferation (signs of traction bronchiectasis or reticular opacities).

**Results:**

Among the 153 patients, 72 (47%) were successfully liberated from ECMO in the short term, while short-term liberation failed in the remaining 81 (53%) patients. Multivariate logistic regression analysis showed that the presence of mixed-pattern pulmonary opacities and signs of traction bronchiectasis, but not the distribution of the opacities, were independently associated with difficulty in short-term liberation (OR [95% CI]; 4.8 [1.4–16.5] and 3.9 [1.4–11.2], respectively).

**Conclusions:**

The presence of a mixed pattern of the pulmonary opacities and signs of traction bronchiectasis on the chest CT were independently associated with difficulty in short-term liberation from V-V ECMO in severe ARDS patients.

**Supplementary Information:**

The online version contains supplementary material available at 10.1186/s12931-023-02425-2.

## Background

Respiratory support using veno-venous extracorporeal membrane oxygenation (V-V ECMO) is an effective strategy for improving the arterial oxygen saturation in patients with severe acute respiratory distress syndrome (ARDS) receiving mechanical ventilation and has the potential for improving the patient outcomes [1–3]. However, analysis of data from a nationwide registry reported that despite use of ECMO where indicated, the hospital mortality remained high at 54.4% [4], indicating that we need to develop better strategies for management of V-V ECMO to improve the patient outcomes.

Prolonged use of ECMO support for patients with ARDS has become more common in the last decade [5]. Considering that long-term ECMO support requires huge amounts of medical and human resources, and may also be associated with many kinds of complications such as bleeding, infection, and muscle weakness [6], it is clinically important to estimate, even at the time of initiation of the support, the difficulty of liberation from ECMO in the short term. It may be a better option to consider immediate transportation of patients in whom liberation from ECMO in the short term is likely to be difficult to the highest volume centers in the country. However, no tool has been established yet for predicting the difficulty in liberation from V-V ECMO in the short term.

ARDS is a heterogeneous syndrome [7, 8], and the characteristics of “bilateral opacities”, based on the Berlin criteria of ARDS [9], are different in each individual patient [10–12]. The characteristics of the pulmonary opacities on chest CT are crucial for understanding the pathophysiology of ARDS [13, 14], but there is no study to investigate the characteristics of the opacities on chest CT in patients with severe ARDS requiring ECMO. We hypothesized that we might be able to predict the likelihood of difficulty in short-term liberation from V-V ECMO based on the chest CT findings at the time of initiation of the support. Therefore, the aim of this study was to identify the characteristics of the pulmonary opacities on chest CT associated with difficulty in liberation from V-V ECMO in the short term.

## Methods

### Study design and population

The study included the data of all adult patients (18 years or older) admitted to any of four intensive care units (ICUs) in Japan (named below) who were initiated on V-V ECMO support for severe ARDS between January 2012 and June 2022. All four ICUs, Hiroshima University Hospital, Japan Red Cross Maebashi Hospital, Nagoya University Hospital, and Tsukuba Memorial Hospital are teaching hospitals and have treated more than 10 patients requiring ECMO per year. The diagnosis of severe ARDS was made based on the Berlin definition criteria (PaO2/FiO2 ratio [P/F ratio] < 100 mmHg) [9]. Patients were excluded if they were cases of conversion from initial veno-arterial (V-A) ECMO or had not undergone chest CT examination at the time of initiation of the ECMO support (within 3 days of initiation). The study was conducted with the approval of the Institutional Review Boards of Hiroshima University Hospital, which waived the requirement for obtaining informed patient consent from the study participants to ensure participant anonymity, as stipulated in the Japanese government guidelines.

Data, including patient demographic information, comorbidities, etiology of ARDS, laboratory test results, chest CT images, clinical course after admission, and outcome were collected retrospectively from the electronic medical charts. The sequential organ failure assessment (SOFA) score was calculated at the time of initiation of the ECMO support as a scale of the illness severity [15].

### Outcomes

The primary outcome measured was difficulty in liberation from V-V ECMO within a short period of time. We defined short-term liberation from ECMO as liberation within 14 days (< 14 days) of initiation of the support [5, 16]. Patients who died before liberation or who were only liberated in the long term (≥ 14 days) were classified as the difficulty in short-term liberation from ECMO group (dsECMO group), while those who were successfully liberated in the short-term were classified into the successful short-term liberation from ECMO group (sECMO group). For patients who needed re-cannulation for ECMO due to recurrent deterioration of the clinical condition, the duration of the ECMO run were calculated by adding the first and second periods of ECMO support. The secondary outcome measured was in-hospital mortality.

### Patient management before/during ECMO and weaning from ECMO

The patients were managed in accordance with the guidelines [17, 18]. We mainly employed protective lung ventilation (≤ 6 ml /ideal body weight and plateau pressure ≤ 30 cmH_2_O) for the patients prior to the initiation of V-V ECMO support. Respiratory support by using ECMO was considered if the patients had hypoxemic respiratory failure with P/F ratio < 150 mmHg on high FiO2 > 0.9 and optimized positive end-expiratory pressure (PEEP). While initiating ECMO support, the support of the invasiveness of mechanical ventilation was reduced for lung protection. The preset goals for oxygenation were a PaO2 of 55–65 mm Hg. Accordingly, the tidal volume was decreased so that the plateau pressure did not exceed 30 cm H_2_O. After improvement of the lung function, the extracorporeal blood flow rate was reduced stepwise to 2.0 L per min. Thereafter, the gas flow was tapered and finally switched off typically for 2–8 h. If the arterial blood gas and respiratory parameters remained stable, the ECMO system was removed.

### Interpretation of chest CT

The pulmonary opacities on the chest CT were interpreted by a specialist radiologist (WF) and a specialist intensivist (MN) in a blinded manner, both of whom had more than 10 years’ experience in interpreting chest CT images of patients with ARDS. The concordance rates between the two evaluators are summarized in supplementary Table 1. We confirmed that the concordance rates were acceptable based on a previous report (kappa statistic ≥ 0.4) [19]. Any disagreement was resolved by review by a third blinded specialist in pulmonary medicine (SO).

The pulmonary opacities were evaluated for the following three characteristics: (1) distribution (focal/diffuse on the dorso-ventral axis or unilateral/bilateral on the left-right axis); (2) intensity (pure ground glass/pure consolidation/mixed); and (3) degree of fibroproliferation (signs of traction bronchiectasis or reticular opacities), based on previous reports [20, 21]. The definitions of these findings were based on a reference [22].

### Statistical analysis

Chi-squared test and Mann-Whitney’s U test were used to compare categorical and continuous variables, respectively. To identify the chest CT findings associated with difficulty in short-term liberation, we performed multivariate logistic regression analysis with adjustments for 4 variables, including the age, the underlying cause of ARDS, and SOFA score at ECMO initiation as a scale of the disease severity, as well as the interval between the start of initiation of mechanical ventilation and ECMO support (> 7 days vs. ≤ 7 days), which showed a statistical significance in the univariate analysis. All reported P values were two-sided, and P < 0.05 was regarded as denoting statistically significant difference. All analyses were conducted using the JMP Pro software (version 16.0, SAS Institute Inc.)

## Results

**Fig. 1:**
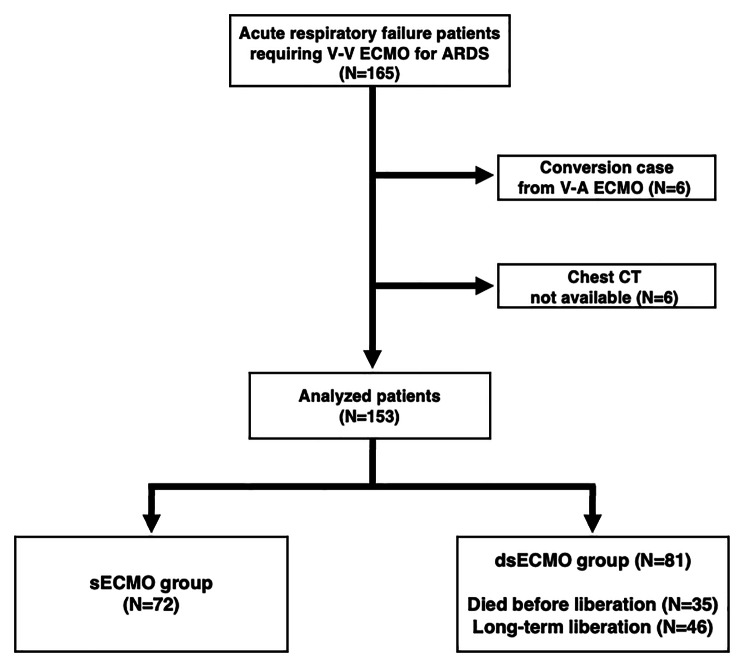
Flow diagram of patients

A total of 165 severe ARDS patients who received V-V ECMO support were included. Of these, 12 patients were excluded as they had been converted from V-A ECMO to V-V ECMO (n = 6) or had not undergone chest CT examination at the time of start of the V-V ECMO support (n = 6), and the data of the remaining 153 patients were analyzed in this study (Fig. 1). Of the 153 patients, 72 (47.1%) were classified into the sECMO group, while the remaining 81 (52.9%) were classified into the dsECMO group (including 35 [22.9%] who died before liberation, and 46 [30.1%] who were liberated in the long term). The characteristics of the analyzed patients, such as the age, sex, and comorbidities, are shown in Table 1. The time difference between the chest CT examinations and start of the V-V ECMO support are shown in supplementary Fig. 1; as shown, the CT examination was performed within 1 day of the start of the ECMO support in the majority of the patients included in the analysis (83.7% [128/153]).

**Table 1:** Baseline characteristics of all subjects

	sECMO group (n = 72)	dsECMO group (n = 81)	P
Age, y	60.5 (49.0–69.0)	65.0 (53.5–71.5)	0.06
Sex, male, *n* (%)	56 (77.8)	64 (79.0)	> 0.99
BMI, kg/m^2, a^	23.6 (21.8–29.0)	24.8 (22.4–28.3)	0.38
Past medical history			
Hypertension, *n* (%)	23 (31.9)	32 (39.5)	0.40
Diabetes, *n* (%)	21 (29.2)	18 (22.2)	0.36
Chronic kidney disease, *n* (%)	8 (11.1)	6 (7.4)	0.58
COPD, *n* (%)	6 (8.3)	7 (8.6)	> 0.99
Asthma, *n* (%)	3 (4.2)	4 (4.9)	> 0.99
Interstitial lung diseases, *n* (%)	5 (6.9)	6 (7.4)	> 0.99
Lung cancer, *n* (%)	3 (4.2)	7 (8.6)	0.34
Chronic heart failure, *n* (%)	3 (4.2)	6 (7.4)	0.50
Interval MV-ECMO > 7 days, *n* (%)	4 (5.6)	13 (16.1)	0.04
Primary reason for ARDS, *n* (%)			0.11
Pneumonia	58 (80.6)	68 (84.0)	
Bacterial	28 (38.9)	19 (23.5)	
Influenza	9 (12.5)	6 (7.4)	
COVID-19	11 (15.3)	20 (24.7)	
Others	10 (13.9)	23 (28.4)	
Extra-pulmonary	5 (6.9)	6 (7.4)	
Drowning	5 (6.9)	5 (6.2)	
Trauma	4 (5.6)	2 (2.5)	
Use of muscle relaxants before ECMO support, *n* (%)	27 (37.5)	36 (44.4)	0.41
Prone positioning before ECMO support, *n* (%)	5 (6.9)	10 (12.4)	0.29
SOFA score at ECMO instauration	12.5 (11.0–14.0)	13.0 (12.0–15.0)	0.10
PF ratio just before ECMO instauration	70.9 (57.8–90.0)	74.0 (55.4–95.6)	0.59
PEEP at the timing of CT examination, cmH_2_O	10 (8–12)	10 (10–14)	0.13
Use of steroid within the first 2 weeks of ECMO support, *n* (%)	35 (48.6)	48 (60.0)	0.19
Use of muscle relaxants within the first 48 h of ECMO support, *n* (%)	32 (44.4)	44 (54.3)	0.26
Prone positioning within the first 2 weeks of ECMO support, *n* (%)	3 (4.2)	4 (4.9)	> 0.99
Duration of ECMO run, days	7.0 (5.3-9.0)	19.0 (15.0-28.5)	< 0.001
Re-canulation of ECMO, *n* (%)	0 (0.0)	11 (13.6)	< 0.001
Mortality at 14 days after ECMO support, *n* (%)	0 (0.0)	8 (9.9)	0.007
Mortality at hospital discharge, *n* (%)	9 (12.5)	49 (60.5)	< 0.001

Data are presented as the median and interquartile ranges (25–75% percentile), or as absolute frequencies with percentages

Abbreviations: sECMO group, successful short-term liberation from ECMO group; dsECMO group, difficulty in short-term liberation from ECMO group; BMI, body mass index; COPD, chronic obstructive pulmonary disease; MV, mechanical ventilation; ECMO, extracorporeal membrane oxygenation; ARDS, acute respiratory distress syndrome; COVID-19, coronavirus disease 2019; SOFA score, sequential organ failure assessment score; PF ratio, PaO_2_/F_I_O_2_ ratio; PEEP, positive end-expiratory pressure

^a^Missing value = 3

**Fig. 2:**
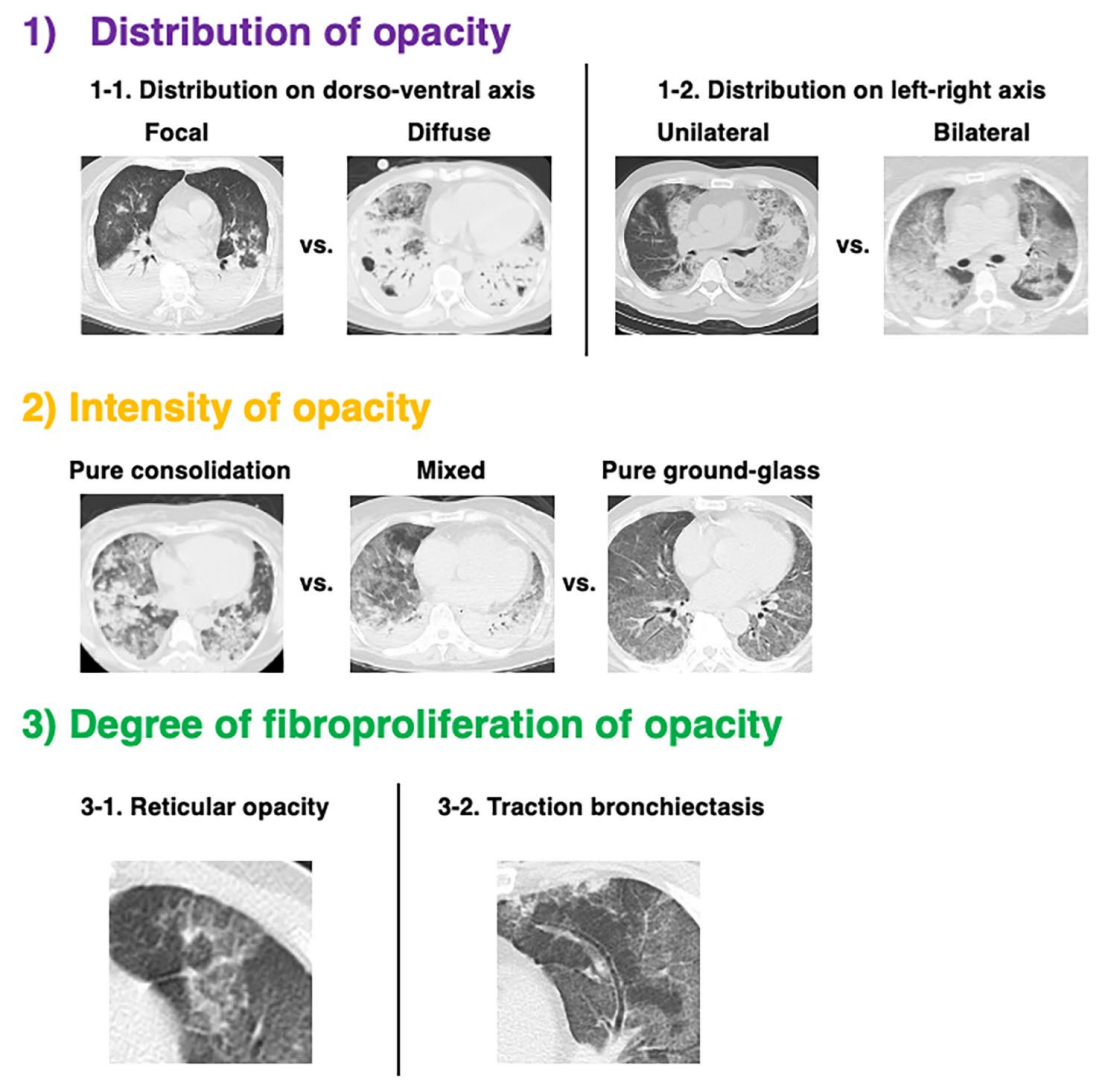
Typical images for each of the characteristic pulmonary opacities on chest CT

The interpretations of the pulmonary opacities on the chest CT are summarized in Table 2, and typical images for each finding are shown in Fig. 2. The characteristics of the pulmonary opacities on chest CT according to the underlying etiology of ARDS were shown in supplementary Fig. 2. Multivariate analysis identified the mixed pattern of pulmonary opacities, as compared with the pure consolidation pattern, and signs of traction bronchiectasis, but not the distribution of the opacities, as being independently associated with difficulty in liberation from the V-V ECMO in the short term (OR [95% CI]; 4.8 [1.4–16.5] and 3.9 [1.4–11.2], respectively) (Table 3). As sensitivity analysis, by using the data of 118 patients who were successfully liberated from ECMO (excluding 35 patients who died before liberation), we also performed multivariate analysis for difficult short-term liberation from ECMO, which confirmed that the mixed pattern of pulmonary opacities and signs of traction bronchiectasis were significantly associated with difficulty in short-term liberation (supplementary Table 2).

**Table 2:** **Characteristics of pulmonary opacity on chest CT between two groups**

	All n = 153	sECMO n = 72	dsECMO n = 81	P
Distribution of opacity				
Distribution on dorso-ventral axis, diffuse (vs. focal), *n* (%)	125 (81.7)	60 (83.3)	65 (80.3)	0.68
Distribution on left-right axis, bilateral (vs. unilateral), *n* (%)	133 (86.9)	60 (83.3)	73 (90.1)	0.24
Intensity of opacity				0.004
Pure consolidation, *n* (%)	64 (41.8)	40 (55.6)	24 (29.6)	
Mixed, *n* (%)	40 (26.1)	13 (18.1)	27 (33.3)	
Pure ground-glass, *n* (%)	49 (32.0)	19 (26.4)	30 (37.0)	
Degree of fibroproliferation of opacity				
Reticular opacity, *n* (%)	79 (51.6)	33 (45.8)	46 (56.8)	0.20
Traction bronchiectasis, *n* (%)	43 (28.1)	11 (25.6)	32 (74.4)	0.001

Data are presented as absolute frequencies with percentages

sECMO group, successful short-term liberation from ECMO group; dsECMO group, difficulty in short-term liberation from ECMO group

**Table 3:** Multivariate analysis for difficult short-term liberation from V-V ECMO

Variable	OR (95%CI)	P
Age	1.02 (0.99–1.05)	0.138
Interval MV-ECMO > 7 days	2.22 (0.60–8.18)	0.231
Primary reason for ARDS		
Pneumonia		
Bacterial	Reference	
Influenza	0.92 (0.24–3.58)	0.908
COVID-19	3.18 (0.96–10.61)	0.059
Others	3.12 (1.02–9.55)	0.047
Extra-pulmonary	2.18 (0.45–10.52)	0.332
Drowning	2.34 (0.47–11.69)	0.299
Trauma	1.63 (0.15–17.26)	0.684
SOFA score at ECMO instauration	1.11 (0.94–1.31)	0.221
Characteristics of opacity		
Distribution of opacity		
Distribution on dorso-ventral axis, diffuse (ref: focal)	0.42 (0.13–1.37)	0.150
Distribution on left-right axis, bilateral (ref: unilateral)	1.87 (0.59–5.97)	0.289
Intensity of opacity		
Pure consolidation	Reference	
Mixed	4.82 (1.41–16.47)	0.012
Pure ground-glass	2.33 (0.74–7.38)	0.149
Degree of fibroproliferation of opacity		
Reticular opacity	0.50 (0.18–1.41)	0.191
Traction bronchiectasis	3.94 (1.38–11.24)	0.010

Abbreviations: MV, mechanical ventilation; ECMO, extracorporeal membrane oxygenation; ARDS, acute respiratory distress syndrome; COVID-19, coronavirus disease 2019; SOFA score, sequential organ failure assessment score; ref reference

We also evaluated the associations between the characteristics of the pulmonary opacities and the mortality at hospital discharge. Multivariate logistic regression analysis showed that none of the characteristics of the opacities was associated with the in-hospital mortality, including the intensity of the opacities and signs of traction bronchiectasis, both of which were associated with difficulty in short-term liberation from ECMO (Table 4).

**Table 4:** Multivariate analysis for in-hospital mortality

Variable	OR (95%CI)	P
Age	1.02 (0.99–1.05)	0.318
Interval MV-ECMO > 7 days	0.48 (0.13–1.71)	0.256
Primary reason for ARDS		
Pneumonia		
Bacterial	Reference	
Influenza	0.09 (0.01–0.78)	0.029
COVID-19	0.50 (0.15–1.65)	0.254
Others	1.95 (0.68–5.65)	0.216
Extra-pulmonary	1.89 (0.40–8.86)	0.417
Drowning	0.14 (0.01–1.38)	0.092
Trauma	1.10 (0.12–10.30)	0.933
SOFA at ECMO instauration	1.18 (1.00-1.39)	0.040
Characteristics of opacity		
Distribution of opacity		
Distribution on dorso-ventral axis, diffuse (ref: focal)	0.52 (0.17–1.63)	0.261
Distribution on left-right axis, bilateral (ref: unilateral)	1.96 (0.62–6.27)	0.254
Intensity of opacity		
Pure consolidation	Reference	
Mixed	1.27 (0.38–4.22)	0.693
Pure ground-glass	1.08 (0.32–3.68)	0.902
Degree of fibroproliferation of opacity		
Reticular opacity	1.86 (0.64–5.38)	0.255
Traction bronchiectasis	1.92 (0.71–5.21)	0.200

Abbreviations: MV, mechanical ventilation; ECMO, extracorporeal membrane oxygenation; ARDS, acute respiratory distress syndrome; COVID-19, coronavirus disease 2019; SOFA score, sequential organ failure assessment score; ref reference

## Discussion

In this retrospective study, we found, in regard to the characteristics of the pulmonary opacities in patients with severe ARDS, that the presence of a mixed pattern of pulmonary opacities, as compared with a pure consolidation pattern, and signs of traction bronchiectasis, but not the distribution of the pulmonary opacities, were independently associated with difficulty in liberation from V-V ECMO in the short term. We believe that this study is the first to investigate the characteristics of pulmonary opacities on the chest CT in patients with severe ARDS requiring V-V ECMO support.

Many previous studies have reported the existence of a strong relationship between the chest CT findings and the etiopathology of ARDS [13, 14]. Although it can also be seen in the early phase [23], fibrosis is one of the major characteristics in the late phase of the pathology of ARDS [13, 14] that is linked to the need for prolonged mechanical ventilatory support as well as to worse outcomes [1]. The reason for the significant association of signs of traction bronchiectasis on imaging and short-term liberation difficulty from ECMO, is that this finding may be a reliable index of the degree of fibroproliferation in cases of severe ARDS. Interestingly, the presence of reticular opacities was not associated with the patient outcomes, although it is also regarded as an index of the degree of fibroproliferation; a possible explanation is that as compared with traction bronchiectasis, reticular opacities also represent many kinds of radiological changes, including interlobular septal thickening, intralobular interstitial thickening, and peri-bronchovascular interstitial thickening, which can also be observed in non-fibrotic areas [24].

The mixed pattern of pulmonary opacities was found to be independently associated with an increased risk of short-term liberation from ECMO, as compared with pure consolidation and pure ground-glass opacities. One possible reason is that in the pathology of ARDS, with progression to/of the fibroproliferative phase, the lung volumes shrink, which can lead to an increase in the density of some regions of the lungs in which ground-glass opacities are observed [20]. The intensity of the mixed-pattern opacities may be a marker of progression to the fibroproliferative phase in patients with severe ARDS, similar to the signs of traction bronchiectasis. Another possible reason is that mixed-pattern opacities may be a sign suggesting that the etiology of the severe ARDS is not simple, such as pure bacterial pneumonia (typically pure consolidation) or pure viral pneumonia (typically pure ground-glass), but complex, such as combined bacterial and viral pneumonia, which may necessitate a longer duration of treatment. But further studies to investigate the mechanism underlying the association of mixed-pattern opacities on chest CT with an increased risk of difficulty in libeartion from ECMO in the short term are needed.

On the other hand, to our surprise, no characteristics of the pulmonary opacities on CT were associated with the in-hospital mortality in patients with severe ARDS requiring ECMO after adjustments for confounding factors. Given that some underlying causes of ARDS were found to be independently associated with the patient mortality in our study, consistent with several previous reports as well [25, 26], the etiology of ARDS, rather than the characteristics of the pulmonary opacities, may be a more important determinant of the risk of patient mortality. Our results also suggest the possibility that an increased risk of short-term liberation difficulty from ECMO may not necessarily be associated with an increased risk of mortality, which may imply that these patients can still be saved by appropriate management of ECMO.

There were several limitations of our study. First, even though our study was a retrospective study conducted based on the data obtained from a heterogeneous population of patients admitted to 4 participating hospitals, an even larger multicenter study is still needed. Second, the values of PEEP at the time of the chest CT examination were varied. PEEP-induced alveolar recruitment can transform poorly aerated lung areas into normally aerated lung areas, potentially influencing the results of the interpretations in this study [27]. Although we confirmed the absence of any statistical significant association between the values of PEEP at the time of the chest CT examination and interpretation of the chest CT findings (unpublished data), a prospective study in which the PEEP setting at the time of the CT examination is kept fixed in all the analyzed patients included in the study would be needed to confirm this finding. Third, in this study, as we did not adopt prone positioning during V-V ECMO support in the majority of patients (7/153, 4.6%), we could not adequately evaluate the influence of prone positioning on the duration of ECMO support. However, we do believe that it would be of great interest to investigate this in the near future. Finally, lung transplantation is rarely performed in Japan (none of the cases in this study underwent transplantation), and the management during ECMO support could be different in patients being considered for lung transplantation.

## Conclusions

The presence of a mixed pattern of the pulmonary opacities and signs of traction bronchiectasis on the chest CT, but not the distribution of the opacities, were independently associated with difficulty in short-term liberation from V-V ECMO in severe ARDS patients.

## Electronic supplementary material

Below is the link to the electronic supplementary material.

Supplementary Table 1: Concordance rates between two evaluators

Supplementary Table 2: Multivariate analysis for difficult short-term liberation in the patients who were successfully liberated from ECMO

Supplementary Figure 1: Time difference between the chest CT examinations and start of V-V ECMO support

Supplementary Figure 2: Characteristics of the pulmonary opacities on chest CT according to the underlying etiology of the acute respiratory distress syndrome

## Data Availability

The datasets used and analyzed during the current study are available from the corresponding author upon reasonable request.

## List of abbreviations

V-V ECMO: Veno-venous extracorporeal membrane oxygenation
ARDS: Acute respiratory distress syndrome
ICUs: Intensive care units
P/F ratio: PaO2/FiO2 ratio
V-A ECMO: Veno-arterial extracorporeal membrane oxygenation
SOFA score: Sequential organ failure assessment score
dsECMO group: Difficulty in short-term liberation from ECMO group
sECMO group: Successful short-term liberation from ECMO group
PEEP: Positive end-expiratory pressure
