# Supplementary material for: Characteristics of the pulmonary opacities on chest CT associated with difficulty in short-term liberation from veno-venous ECMO in patients with severe ARDS
Source: Respir Res. 2023 May 10;24:128. doi: 10.1186/s12931-023-02425-2 (PMC10171155; doi:10.1186/s12931-023-02425-2)
Supplement: Supplementary file 1 — Supplementary Table 1: Concordance rates between two evaluators [file 12931_2023_2425_MOESM1_ESM.docx]

**Supplementary Table 1. Concordance rates between two evaluators**

|  | **Kappa statistics (95% CI)** |
| --- | --- |
| Distribution of opacity |  |
| Distribution on dorso-ventral axis | 0.89 (0.79-0.98) |
| Distribution on left-right axis | 0.83 (0.70-0.95) |
| Intensity of opacity | 0.73 (0.64-0.82) |
| Degree of fibroproliferation of opacity |  |
| Reticular opacity | 0.87 (0.79-0.95) |
| Traction bronchiectasis | 0.69 (0.56-0.82) |

Abbreviations: 95% CI 95% confidence interval.
