# Supplementary material for: Characteristics of the pulmonary opacities on chest CT associated with difficulty in short-term liberation from veno-venous ECMO in patients with severe ARDS
Source: Respir Res. 2023 May 10;24:128. doi: 10.1186/s12931-023-02425-2 (PMC10171155; doi:10.1186/s12931-023-02425-2)
Supplement: Supplementary file 2 — Supplementary Table 2: Multivariate analysis for difficult short-term liberation in the patients who were successfully liberated from ECMO [file 12931_2023_2425_MOESM2_ESM.docx]

**Supplementary Table 2. Multivariate analysis for difficult short-term liberation in the patients who were successfully liberated from ECMO**

| **Variable** | **OR (95%CI)** | **P** |
| --- | --- | --- |
| Age | 1.02 (0.97-1.07) | 0.442 |
| Interval MV-ECMO > 7 days | 7.47 (1.50-37.1) | 0.014 |
| Primary reason for ARDS |  |  |
| Pneumonia |  |  |
| Bacterial | Reference |  |
| Influenza | 3.22 (0.62-16.82) | 0.165 |
| COVID-19 | 6.06 (1.33-27.55) | 0.020 |
| Others | 5.56 (1.22-25.24) | 0.026 |
| Extra-pulmonary | 1.53 (0.15-15.91) | 0.724 |
| Drowning | 9.60 (1.33-69.40) | 0.025 |
| Trauma | 1.35 (0.03-56.53) | 0.874 |
| SOFA score at ECMO instauration | 1.10 (0.90-1.35) | 0.341 |
| Characteristics of opacity |  |  |
| Distribution of opacity |  |  |
| Distribution on dorso-ventral axis, diffuse (ref: focal) | 0.32 (0.06-1.74) | 0.188 |
| Distribution on left-right axis, bilateral (ref: unilateral) | 7.01 (0.87-55.13) | 0.067 |
| Intensity of opacity |  |  |
| Pure consolidation | Reference |  |
| Mixed | 7.04 (1.48-33.46) | 0.014 |
| Pure ground-glass | 3.05 (0.71-13.17) | 0.135 |
| Degree of fibroproliferation of opacity |  |  |
| Reticular opacity | 0.40 (0.11-1.44) | 0.160 |
| Traction bronchiectasis | 3.64 (1.00-13.18) | 0.049 |

Abbreviations: MV, mechanical ventilation; ECMO, extracorporeal membrane oxygenation; ARDS, acute respiratory distress syndrome; COVID-19, coronavirus disease 2019; SOFA score, sequential organ failure assessment score; ref reference.
