# Supplementary figures and images for: Characteristics of the pulmonary opacities on chest CT associated with difficulty in short-term liberation from veno-venous ECMO in patients with severe ARDS
Source: Respir Res. 2023 May 10;24:128. doi: 10.1186/s12931-023-02425-2 (PMC10171155; doi:10.1186/s12931-023-02425-2)

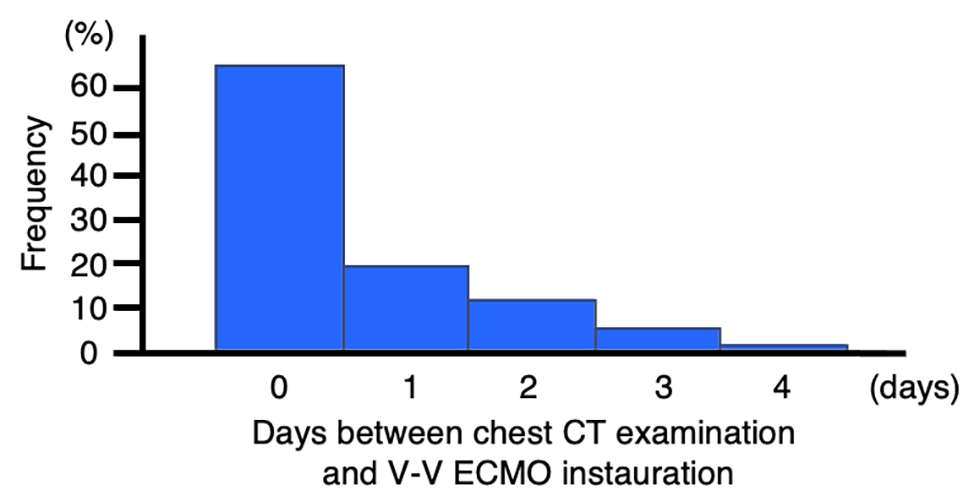

Supplement: Supplementary file 3 — Supplementary Figure 1: Time difference between the chest CT examinations and start of V-V ECMO support [file 12931_2023_2425_MOESM3_ESM.png]

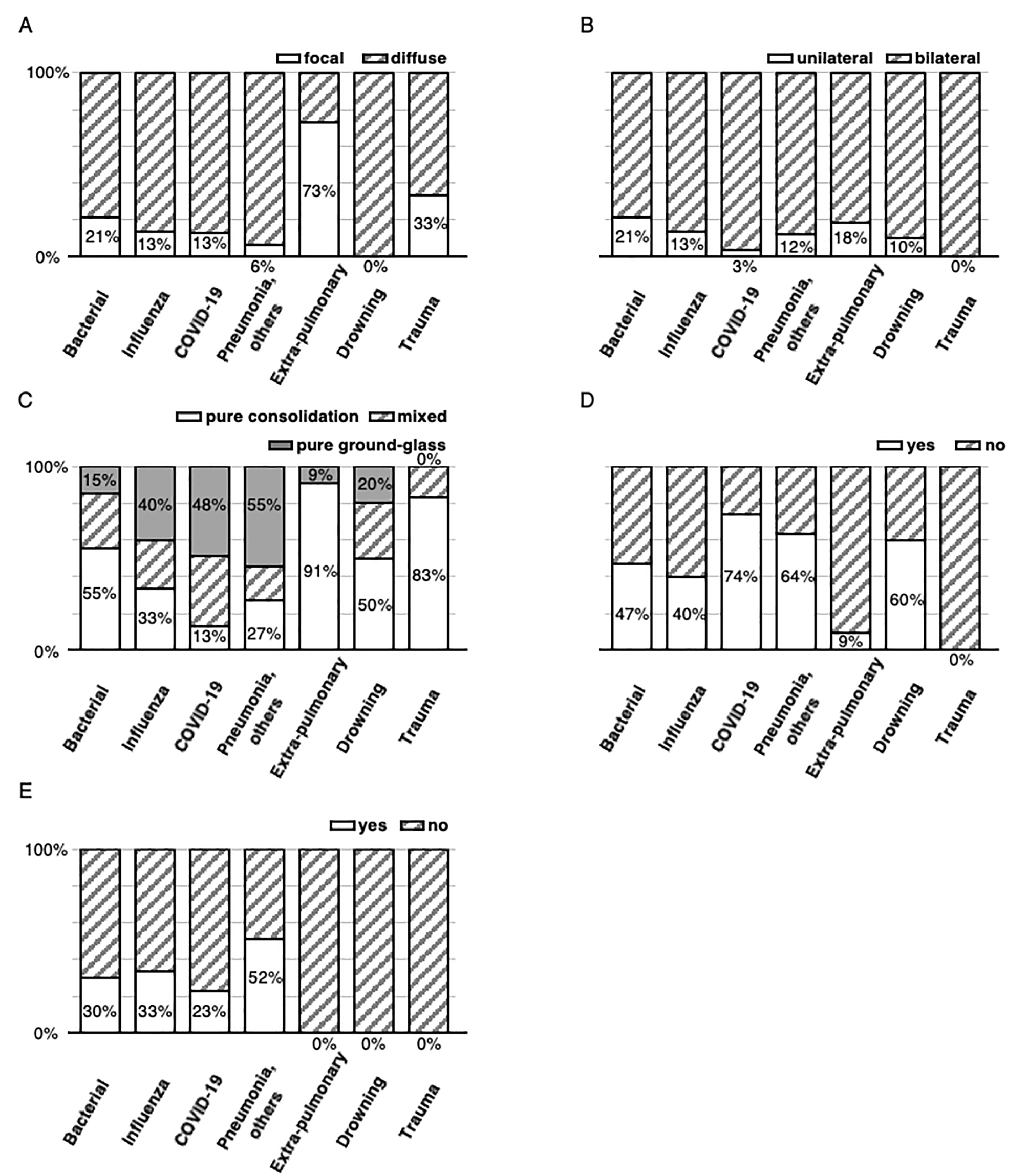

Supplement: Supplementary file 4 — Supplementary Figure 2: Characteristics of the pulmonary opacities on chest CT according to the underlying etiology of the acute respiratory distress syndrome [file 12931_2023_2425_MOESM4_ESM.png]
